# Functional Comparison for Lipid Metabolism and Intestinal and Fecal Microflora Enzyme Activities between Low Molecular Weight Chitosan and Chitosan Oligosaccharide in High-Fat-Diet-Fed Rats

**DOI:** 10.3390/md15070234

**Published:** 2017-07-24

**Authors:** Chen-Yuan Chiu, Shih-An Feng, Shing-Hwa Liu, Meng-Tsan Chiang

**Affiliations:** 1 Department of Cell and Tissue Engineering, Changhua Christian Hospital, Changhua 500, Taiwan; kidchiou@gmail.com; 2 Institute of Toxicology, College of Medicine, National Taiwan University, No. 1, Section 1, Jen-Ai Rd., Taipei 100, Taiwan; 3 Department of Food Science, College of Life Science, National Taiwan Ocean University, Keelung 202, Taiwan; afrankgod@gmail.com; 4 Department of Pediatrics, College of Medicine and Hospital, National Taiwan University, Taipei 100, Taiwan; 5 Department of Medical Research, China Medical University Hospital, China Medical University, Taichung 404, Taiwan

**Keywords:** low molecular weight chitosan, chitosan oligosaccharide, lipid metabolism, fecal mucinase, fecal β-glucuronidase

## Abstract

The present study investigated and compared the regulatory effects on the lipid-related metabolism and intestinal disaccharidase/fecal bacterial enzyme activities between low molecular weight chitosan and chitosan oligosaccharide in high-fat-diet-fed rats. Diet supplementation of low molecular weight chitosan showed greater efficiency than chitosan oligosaccharide in suppressing the increased weights in body and in liver and adipose tissues of high-fat-diet-fed rats. Supplementation of low molecular weight chitosan also showed a greater improvement than chitosan oligosaccharide in imbalance of plasma, hepatic, and fecal lipid profiles, and intestinal disaccharidase activities in high-fat-diet-fed rats. Moreover, both low molecular weight chitosan and chitosan oligosaccharide significantly decreased the fecal microflora mucinase and β-glucuronidase activities in high-fat-diet-fed rats. These results suggest that low molecular weight chitosan exerts a greater positive improvement than chitosan oligosaccharide in lipid metabolism and intestinal disaccharidase activity in high-fat-diet-induced obese rats.

## 1. Introduction

Accompanying the global change in dietary consumption with western-style foods, there has been an increasing trend towards the development of metabolic syndrome-related disorders by the presence of an energy imbalance, such as obesity and diabetes mellitus (DM) [1]. The worldwide prevalence of obesity has more than doubled between 1980 and 2014 [2]. On the other hand, the global prevalence of DM among adults over 18 years of age has been elevated from 4.7% in 1980 to 8.5% in 2014 [3]. Moreover, the term “diabesity” has been coined with the interdependent relationship between obesity and diabetes [4], especially type 2 DM (T2DM). Obesity and T2DM frequently occur together with the characteristics of dysregulating lipid metabolism and insulin resistance [5]. Field et al. (2001) have suggested that the relative risk for female and male obese patients to develop T2DM is 10-fold and 11.2-fold, respectively [6]. Hence, the strategy to regulate homeostasis of lipid metabolism and glucose metabolism is concerned with anti-obesity food and food ingredients against human diabesity.

Nutraceutical interventions referred to as ‘functional foods’ are currently being investigated as potential strategies to prevent or ameliorate the development of obesity and its co-morbidities—such as cardiovascular disease, stroke, and diabetes—by assisting the body energy homeostasis mechanisms [7]. Recently, chitosan and its derivatives, known as marine functional foods, are a partially deacetylated form of chitin composed of *N*-acetyl-d-glucosamine from marine organisms [8,9], which can be applied to act as the dietary fiber against metabolic disorders due to its viscous and indigestible nature [10,11,12]. Human studies have shown that chitosan displays a dual face in body weight reduction of obese participants in clinical trials [13,14,15,16], these facts may be suspected due to various characteristics of chitosan, including its molecular weight and its derivatives. According to its characteristics of biocompatibility and nontoxic nature, there has recently been a growing interest on the therapeutic potential of the depolymerized derivatives of low molecular weight chitosan (LC) and chitosan oligosaccharides (CO) against obesity, DM, or other metabolic disorders [17,18]. However, the comparison of therapeutic potentials on functional properties of regulating lipid metabolism and intestinal/fecal enzyme activities between LC and CO is not well documented. Therefore, in the present study, high-fat(HF)-diet-induced obese rats were used as an in vivo metabolic imbalance model to clarify the mode of action of various functional differences between LC and CO in regulating lipid metabolism and intestinal disaccharidase/fecal bacterial enzyme activities.

## 2. Materials and Methods

### 2.1. Materials

LC and CO from crab shell were purchased from Koyo Chemical Co. (Tokyo, Japan). The concoction of chitosan was processed from the crab shell with demineralization, deacetylation, and deproteinization. The degree of deacetylation and average molecular weight of chitosan were evaluated by Fourier transform infrared spectroscopy and high-performance liquid chromatography, respectively. The degree of deacetylation and the average molecular weight of chitosan are about 83.9% and 80 kDa, respectively. The degree of deacetylation and the average molecular weight of chitosan oligosaccharides are 100% and about 719 kDa, respectively.

### 2.2. Animals and Diets

Male Sprague–Dawley (SD) rats aged six weeks from BioLASCO Taiwan Co., Ltd. (Taipei, Taiwan) were domesticated for one week with the normal chow diet (Rodent Laboratory Chow, Ralston Purina, St. Louis, MO, USA). Domestic rats were randomly separated into four groups (*n* = 8 of each group): (1) standard rodent diet-fed rats (NC), (2) HF-diet-fed rats, (3) HF-diet-fed rats with 5% low molecular weight chitosan (HF + LC), and (4) HF-diet-fed rats with 5% chitosan oligosaccharide (HF + CO). The formulation of the experimental diets was displayed in Table 1. All rats housed individually in stainless-steel cages were maintained in temperature- (23 ± 1 °C), light- (12 h light/dark cycle), and humidity-controlled (40–60% relative humidity) rooms. The body weight was measured weekly until sacrifice (10th week). After 10 weeks of experimental intervention, the animals were fasted for 12 h and then sacrificed under anesthesia by exsanguination. Blood samples were harvested for the following biochemical analysis. The collective liver tissues were shaved, weighed, flash-frozen, and stored at −80 °C until hepatic lipid profile analysis. The mucosa of the small intestine from the ligament of Treitz to the cecum were scraped with a chilled glass slide and stored at −80 °C until intestinal disaccharidase activity analysis. Feces were collected for three consecutive days before euthanasia and stored at −80 °C until fecal lipid content analysis. Fresh feces were also assembled for fecal enzyme analysis as previously described [19]. All the experimental procedures for animals in this study were in accordance with the Guide for the Care and Use of Laboratory Animals [20] and approved by the Animal House Management Committee of the National Taiwan Ocean University (permission number: 104015).

### 2.3. Determination of Triglyceride (TG) and Total Cholesterol (TC)

The levels of TG and TC in the plasma, liver, and feces were measured by enzymatic assay kits (Audit Diagnostics, Cork, Ireland) according to the manufacturer’s instructions. Plasma lipoproteins were isolated as previously described [21]. Briefly, the HDL, VLDL, and LDL of plasma were separated by density gradient ultracentrifugation (194,000× *g* at 10 °C for 3 h) (Hitachi, SP85G, RPL 42T Rotor, Tokyo, Japan), and these lipoproteins were recovered by tube slicing.

### 2.4. Determination of Plasma Tumor Necrosis Factor-α (TNF-α)

Rat enzyme-linked immunosorbent assay (ELISA) kits were used to determine levels of plasma TNF-α (Assay Designs, Inc., Ann Arbor, MI, USA).

### 2.5. Determination of Hepatic Fatty Acid Synthetase (FAS) Activity

Hepatic FAS activity was evaluated as previously described [22], and the activity was determined by the rate of nmole NADPH decrease with normalization of protein concentration.

### 2.6. Determination of Small Intestinal Disaccharidase Activity

The collected mucosa samples from each rat were homogenized in chilled physiological saline and the homogenates were then centrifuged at 1570× g for 10 min as previously described by Yao et al. (2008) [23]. The specific activities of lactase, sucrase, and maltase in the supernatants were determined by respectively measuring the amount of glucose released from lactose, sucrose, and maltose as previously described [24]. The glucose concentration was also measured by the Audit Diagnostics glucose kit (Carrigtwohill, Cork, Ireland).

### 2.7. Determination of Fecal Mucinase and β-Glucuronidase Activities

Mucinase and β-glucuronidase activities were determined by the method of Yao and Chiang (2006) [25]. Values of mucinase and β-glucuronidase activities are expressed as nmole of reducing sugar/min/mg protein and nmole of phenolphthalein/min/mg protein, respectively.

### 2.8. Statistical Evaluation

All results are expressed as the Mean ± Standard Deviation (SD) (*n* = 8). The significant difference between the treated group and the respective control is assessed by one-way analysis of variance (ANOVA) and two-tailed Student’s *t*-test with the SPSS statistical software (Windows version 10.0.7C, SPSS, Chicago, IL, USA).

## 3. Results and Discussion

### 3.1. Effects of Both LC and CO on Body Weight, Organ and Tissue Weights, Food Intake, and Feed Efficiency in HF-Diet-Fed Rats

First of all, we examined the effects of both LC and CO on body weight, organ and tissue weights, food intake, and feed efficiency in HF-diet-fed rats. As shown in Table 2, rats fed with 5% LC for 10 weeks had a significant reduction in final body weight as well as in body weight gain as compared with the HF group (11.04% and 14.71%, respectively, *p* < 0.05). The food utilized efficiency of HF-fed rats supplemented with 5% LC was also significantly lower as compared with the HF group (14.29%, *p* < 0.05). Moreover, liver weights and the adipose tissue weights were significantly increased in HF-fed rats, which can be reversed by supplementation of 5% LC. However, supplementation of 5% CO in HF-fed rats had only a significant decrease in the adipose tissue weight (26.92%, *p* < 0.05), but no obvious reduction in final body weight, body weight gain, and feed efficiency. Previous studies are consistent with our results that supplementation of LC prevented increases in body weight and organ weight induced by feeding a HF diet in mice [18] or genetically obese mice (KK-A^y^ mice) [26]. These results suggested that supplementation of LC had a greater ability to ameliorate HF diet-induced overweight than supplementation of CO. Choi et al. (2012) indicated that HF diet supplemented with 3% CO had a significant reduction in body weight gain and adipocyte size, but the period of administration was required to take at least five months [27]. In the current study, there was no obvious effect on body weight gain, food utilization, and weights of the primary site of lipid and glucose regulation (liver) in HF-diet-fed rats supplemented of 5% CO for 2.5 months, whereas supplementation of 5% LC could significantly ameliorate HF diet-induced deleterious effects. Interestingly, Kim et al. (2014) have indicated that low molecular weight CO (GO2KA1, MW < 1000 Da) compared to high molecular weight CO (GO2KA3, MW > 10,000 Da) showed a greater prevention from obesity and DM with carbohydrate-rich diet in T2DM diabetic animals for seven weeks, [28,29] implying that anti-obesity and anti-diabetes effects of chitosan oligosaccharides may depend on the molecular weight in a shorter administered period.

### 3.2. Effects of Both LC and CO on Hepatic and Fecal Lipid Responses and Lipid-Related Metabolic Changes in HF-Diet-Fed Rats

We next examined the effects of LC and CO on lipid profiles in the plasma, liver, and feces. As shown in Table 3, the rats fed a HF diet for 10 weeks significantly increased plasma LDL-C, VLDL-C, LDL-C + VLDL-C, and TC/HDL-C ratios; elevated plasma TC; and decreased HDL-C and HDL/(LDL-C + VLDL-C) ratios. The increased (TC, VLDL-C, and LDL-C + VLDL-C) plasma lipid metabolism profiles in HF-diet-fed rats were effectively reversed by the supplementation of 5% LC, whereas there was no significantly ameliorated effect of supplementation of 5% CO on dysregulation of plasma cholesterol levels, suggesting that diets supplemented with LC are more effectively than diets supplemented with CO in reversing imbalance of plasma cholesterol levels in HF-diet-fed rats. Unexpectedly, the plasma TG levels in HF group were not significantly changed as compared with the NC group, although supplementation of 5% CO, but not 5% LC, could decrease the plasma TG levels in the HF group. The paradoxical reduction in the plasma TG levels in HF-diet-fed rats is accordant with the previous studies and our studies, which may result from the downregulative expressions of the plasma lipoprotein lipase suppressor (microsomal angiopoietin-like 4), the hepatic TG transporter (triglyceride transfer protein), and the hepatic VLDL-TG secretion enhancer (apolipoprotein E), causing the consequence of TG excretion into the feces or accumulation in the liver [30,31,32,33,34].

On the other hand, supplementation of 5% LC, but not 5% CO, could significantly inhibit an increase in plasma levels of TNF-α in HF-diet-fed rats (Table 3). TNF-α served as an adipocytokine has been shown to damage insulin signaling pathway at the insulin receptor substrate level, causing insulin resistance and the impairment of glucose uptake in adipocytes and myocytes [35]. Moreover, TNF-α has been demonstrated to be capable of regulating lipid/adipocyte metabolism [36]. TNF administration to rats could induce hepatic lipid synthesis which might play a key role in increasing serum triglycerides; this effect of TNF-stimulated hepatic lipogenesis has been further demonstrated to be independent of alteration in insulin [37]. A study has also suggested that streptozotocin/nicotinamide-induced diabetic rats supplemented with 5% LC could significantly decrease plasma adipocytokines levels including TNF-α, leptin, and plasminogen activator inhibitor-1 as compared with diabetic rats fed with normal diets [34]. The finding in the present study that 5% LC could decrease plasma TNF-α levels in HF-diet-fed rats was consistent with aforementioned previous studies.

We next examined the effects of LC and CO on the liver characteristics. As shown in Table 4, rats fed with HF diet for 10 weeks showed a significant increase in hepatic TC and TG levels and a non-significant increase in fatty acid synthetase (FAS) activity which leads to lipid biosynthesis in the liver, whereas these abnormal TC and TG levels and FAS activity in the liver were markedly inhibited in the HF-diet-fed rats supplemented with 5% LC. Supplementation of 5% CO only had a significant reduction in hepatic FAS activity and the non-significant decreases in hepatic TC and TG levels. We also measured the levels of TC and TG in the feces. As shown in Table 5, the feces of HF-diet-fed rats supplemented with 5% LC significantly contained more TC and TG than that of HF-diet-fed rats without LC supplementation. There were no significant changes in fecal TC and TG levels of the HF-diet-fed supplemented with 5% CO group. To date, few reports are available on the anti-obesity effects of LC and CO on the lipid metabolism in the plasma, liver, and feces. Hayashi and Ito (2002) have reported that LC (chitosan lactate average MW: 20,000) given as drinking water for 11 weeks dose-dependently (0.05, 0.2 and 0.8% LC) and markedly reduced the serum TG levels, but not TC levels, in genetically obese diabetic KK-A^y^ mice [26]. In addition, Sumiyoshi and Kimura (2006) have suggested that water-soluble LC (MW: 46 kDa; 300 mg/kg, twice daily) could exhibit the lipid-lowering effects with an increase in fecal TG excretion and a decrease in intestinal dietary lipid absorption in HF-diet-fed mice for 20 weeks [18]. In the present study, we showed that LC could effectively lower plasma and hepatic TC and TG levels and increase fecal TC and TG excretion. These results are consistent with the previous studies that anti-obesity effects of LC may be through improving dysregulation of lipid profile in HF-diet-fed animals. Moreover, some studies have reported that genetically obese diabetic mice (*ob*/*ob* and *db*/*db* mice) and HF-diet-fed mice supplemented with CO for five to six weeks or five months display a marked improvement in plasma and hepatic lipid profiles [27,28,38,39]. These findings are contradictory to our present results that the non-obvious effects of 5% CO on plasma and hepatic lipid profiles may be attributed to using different animal models, routes of administration, and treatment periods.

### 3.3. Effects of Both LC and CO on Intestinal Disaccharidase Activities and Fecal Bacterial Enzyme Activities in HF-Diet-Fed Rats

We next examined the effects of both LC and CO on the activities of intestinal disaccharidases (maltase, lactase, and sucrase) and fecal bacterial enzymes (mucinase and β-glucuronidase) in HF-diet-fed rats. As shown in Table 6, the significant decreases in intestinal maltase, lactase, and sucrase and fecal β-glucuronidase activities were found in the HF-diet-fed rats supplemented with 5% LC as compared with HF group. The fecal mucinase activities were found to be a decreased trend with no significant difference in the HF-diet-fed rats supplemented with 5% LC group as compared with HF group. Supplementation of 5% CO significantly reduced fecal mucinase and β-glucuronidase activities in the HF-diet-fed rats. Several studies indicated that obese or diabetic animals had greater intestinal disaccharidase activities than normal animals [40,41,42]. Many viscous soluble dietary fibers could exhibit gastroparesis and decrease intestinal disaccharidase activities, causing reduction in the plasma glucose by delaying the absorption of glucose and inducing the insulin sensitivity of peripheral tissues [43,44]. There were few studies investigating the effects of chitosan acted as dietary fibers on intestinal disaccharidase activities. Our previous study has shown that high molecular weight chitosan, but not low molecular weight chitosan, significantly decreases intestinal disacchridase activities in STZ-induced diabetic rats [23]. In the present study, the activities of intestinal disaccharidases showed a significant reduction in HF-diet-fed rats supplemented with 5% LC, but not with 5% CO. However, dietary fibers have been reported to not only reduce intestinal disaccharidase activities but also inhibit fecal activities of β-glucuronidase and municase, which could degrade the mucin coat in the gut wall and result in losing protection of colonic mucosal cells from toxic or carcinogenic substances [45,46,47]. In addition, previous studies have shown that rats fed a HF diet have a significantly higher fecal microflora enzyme activities of municase and β-glucuronidase [48,49]. We have investigated the relationship between chitosan and the activities of fecal mucinase and β-glucuronidase and the idea that high molecular weight chitosan could significantly inhibit fecal mucinase and β-glucuronidase activities [25]. We suggested that the inhibitory effect of chitosan on β-glucuronidase activity may result from its lipid-lowering effect. On the other hand, chitosan may elicit an inhibitory effect upon fecal mucinase and be available for fermentation in the distal colon, which may result in a reduction in mucin degradation and a stimulation of mucus secretion within the colon [25]. In the current study, we found the significantly ameliorated effects of supplementation of 5% CO on the fecal mucinase and β-glucuronidase activities in HF-diet-fed rats, whereas supplementation of 5% LC could only display a significant reduction in fecal β-glucuronidase activity and a decreased trend for fecal mucinase activity. It is possible that the movement of LC and CO in the colon may be similar to that of the high molecule weight of chitosan. Nevertheless, these findings imply that LC or CO possesses the ability to reverse the HF diet-induced activities of intestinal disaccharidases or fecal microflora enzymes.

The effects of chitosan on body weight reduction in human clinical trials are controversial. Trivedi et al. (2016) [14] and Schiller et al. (2001) [15] have shown a positive effect of reducing body weight in overweight and obese individuals. On the other hand, Mhurchu et al. (2004) [13] and Ho et al. (2001) [16] have indicated that there was no significant alteration of body weight in obese subjects between placebo and chitosan administration. However, they have not claimed the characteristics of chitosan used in these human studies including molecular weight and its derivatives, which may exhibit different influence on body weight and other biological parameters of obese individuals. Moreover, this fact also motivates us to evaluate the functional effects of low molecular weight chitosan and chitosan oligosaccharide on lipid metabolism and related enzyme activities in high-fat-diet-induced obese rats, which could be applied for results of human clinical trials.

## 4. Conclusions

**Figure 1:**
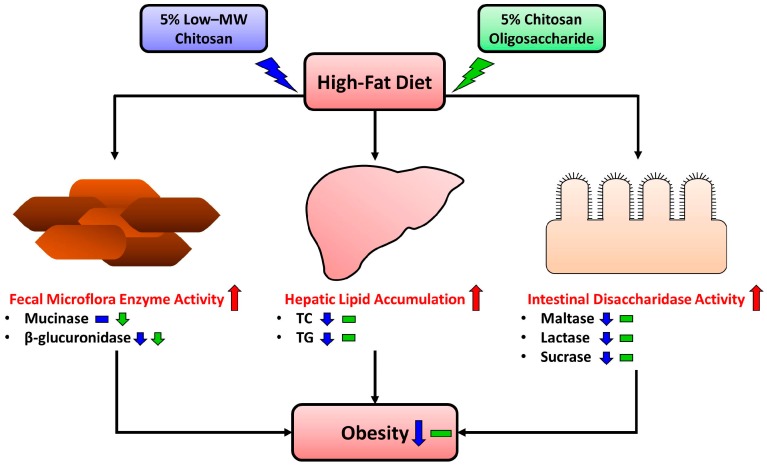
Scheme representing the functional comparison for lipid metabolism and intestinal/fecal enzyme activities between low molecular weight chitosan (LC) and chitosan oligosaccharide (CO) in high-fat (HF)-diet-fed rats. There are the different anti-obesity effects between 5% LC and 5% CO supplementations in HF-diet-fed rats for 10 weeks. LC has relatively greater ability than CO to positively regulate lipid metabolism and intestinal disaccharidase activities in HF-diet-induced obese rats.

The findings of this study suggest that there are the different anti-obesity effects between 5% LC and 5% CO supplementations in HF-diet-fed rats for 10 weeks. LC has relatively greater ability than CO to positively regulate lipid metabolism and intestinal disaccharidase activities in HF diet-induced obese rats (Figure 1). According to the anti-obesity effects of a previous long-term study reported by Choi et al. (2012) [27], it is possible that much more long-term feeding (five months) may be required for CO to reach a significant anti-obesity effect in HF diet-induced obese rats. The detailed effects and mechanisms of both LC and CO on fecal microflora enzyme activities in HF-diet-fed rats still need to be further clarified in the future. The present study may provide valuable information to further clinical trials of low molecular weight chitosan for obese subjects. Moreover, for future studies, the effects of a wider spectrum of molecular weights and deacetylation degrees for chitosan may need to be evaluated, and the influence of mineral- and fat-soluble vitamin status by continuous and massive intake of chitosan may also need to be taking into account.

## Figures and Tables

**Table 1:** Composition of experimental diets (%)

Ingredient	NC ^1^	HF ^2^	HF + LC ^3^	HF + CO ^4^
Lard	-	10	10	10
Cholesterol	-	0.5	0.5	0.5
Cholic acid	-	0.1	0.1	0.1
Chitosan ^5^	-	-	5	-
Chitosan ^6^ oligosaccharide	-	-	-	5
Chow diet	100	89.4	84.4	84.4

^1^ NC: normal control diet; ^2^ HF: high fat diet (10% Lard); ^3^ HF + LC: high fat diet +5% low molecular weight chitosan; ^4^ HF + CO: high fat diet +5% chitosan oligosaccharide; ^5^ The average MW and DD of chitosan are about 8 × 10^4^ Dalton and 83.9%, respectively; ^6^ The average MW and DD of chitosan oligosaccharide are about 719 and 100%, respectively.

**Table 2:** The changes of body weight, liver and perirenal fat weight, food intake, feed efficiency in rats fed the different experimental diets for 10 weeks.

Diet	NC	HF	HF + LC	HF + CO
Initial body weight (g)	158.8 ± 18.4	164.3 ± 12.0	160.4 ± 11.8	162.1 ± 12.6
Final body weight (g)	476.8 ± 21.2 *	550.5 ± 51.7	489.7 ± 49.5 *	495.0 ± 51.9
Body weight gain (g)	318.0 ± 23.0 *	386.2 ± 43.9	329.4 ± 47.7 *	332.9 ± 56.0
Liver weight (g)	12.6 ± 0.6 *	25.5 ± 3.8	19.2 ± 3.7 *^,#^	23.8 ± 3.4
Relative liver weight	-	-	-	-
(g/100 g BW)	2.6 ± 0.1*	4.6 ± 0.4	3.9 ± 0.4 *^,#^	4.9 ± 0.9
Perirenal fat (g)	8.9 ± 2.5*	14.0 ± 4.7	9.9 ± 2.7 *	9.9 ± 1.9 *
Relative perirenal fat weight	-	-	-	-
(g/100 g BW)	1.85 ± 0.5 *	2.6 ± 0.6	1.9 ± 0.5 *	2.0 ± 0.3 *
Food intake (g/day)	30.2 ± 3.1	28.6 ± 3.4	27.5 ± 4.7	28.3 ± 3.4
Feed efficiency (%)	7.3 ± 0.8 *	9.1 ± 1.2	7.8 ± 1.0 *	8.2 ± 1.5

Feed efficiency = (weight gain (g)/food intake (g)) × 100%; Results are expressed as mean ±S.D. for each group rats (*n* = 8); * *p*< 0.05 versus HF; ^#^ *p* < 0.05 versus HF + CO.

**Table 3:** The change of plasma lipid concentrations in rats fed the different experimental diet for 10 weeks.

Diet	NC	HF	HF + LC	HF + CO
Total cholesterol (mg/dL)	52.5 ± 8.3 *	116.9 ± 50.5	73.6 ± 14.9 *	77.2 ± 24.8
HDL-C (mg/dL)	30.6 ± 1.8 *	21.5 ± 11.7	18.2 ± 8.8	13.3 ± 3.6
LDL-C (mg/dL)	16.3 ± 6.4 *	36.6 ± 13.6	28.0 ± 8.5	32.1 ± 17.2
VLDL-C (mg/dL)	5.6 ± 2.8 *	58.8 ± 43.8	27.4 ± 12.2 *	31.8 ± 9.9
LDL-C + VLDL-C	21.9 ± 7.5 *	95.4 ± 49.0	55.4 ± 17.4 *	63.9 ± 26.5
TC/HDL-C ratio	1.7 ± 0.2 *	6.7 ± 5.5	5.1 ± 3.2	6.3 ± 3.1
HDL-C/(LDL-C + VLDL-C) ratio	1.6 ± 0.7 *	0.27 ± 0.2	0.38 ± 0.3	0.25 ± 0.1
Triglyceride (mg/dL)	46.2 ± 19.1	36.3 ± 7.9	29.4 ± 7.7	25.2 ± 6.6 *
TNF-α (pg/mL)	18.5 ± 1.5 *	24.7 ± 1.8	11.4 ± 4.9 *^,#^	25.8 ± 17.0

Results are expressed as mean ±S.D. for each group rats (*n* = 8); * *p* < 0.05 versus HF; ^#^ *p* < 0.05 versus HF + CO.

**Table 4:** The change of hepatic lipid profile and enzyme activity of lipid biosynthesis in rats fed the different experimental diets for 10 weeks.

Diet	NC	HF	HF + LC	HF + CO
Total cholesterol	-	-	-	-
(mg/g liver)	1.5 ± 0.41 *	63.1 ± 10.8	34.7 ± 15.5 *^,#^	67.7 ± 19.4
(g/liver)	0.02 ± 0.01 *	1.60 ± 0.37	0.66 ± 0.30 *^,#^	1.60 ± 0.57
Triglyceride	-	-	-	-
(mg/g liver)	9.8 ± 2.2 *	47.2 ± 15.9	38.8 ± 11.1	38.6 ± 15.9
(g/liver)	0.12 ± 0.03 *	1.20 ± 0.46	0.76 ± 0.31 *	0.93 ± 0.48
Fatty acid synthetase	2.5 ± 2.0	3.3 ± 1.1	2.1 ± 0.9 *	2.2 ± 1.1 *
(nmole NADPH/min/mg protein)				

Results are expressed as mean ±S.D. for each group rats (*n* = 8); * *p* < 0.05 versus HF; ^#^ *p* < 0.05 versus HF + CO.

**Table 5:** The change of fecal weight, triglyceride, and total cholesterol concentration in rats fed the different experimental diets for 10 weeks.

Diet	NC	HF	HF + LC	HF + CO
Feces wet weight (g/day)	9.7 ± 1.7	8.5 ± 2.3	10.7 ± 1.8 *	9.9 ± 1.7
Feces dry weight (g/day)	6.2 ± 0.7	5.7 ± 1.1	6.2 ± 0.6	5.9 ± 0.8
Total cholesterol	-	-	-	-
(mg/g feces)	6.6 ± 1.5 *	12.8 ± 3.1	15.5 ± 1.7 *^,#^	12.9 ± 2.5
(mg/day)	40.6 ± 11.3 *	71.0 ± 23.4	96.5 ± 12.6 *	77.3 ± 22.0
Triglyceride	-	-	-	-
(mg/g feces)	12.2 ± 1.2	12.8 ± 0.9	13.1 ± 0.9	13.6 ± 1.2
(mg/day)	75.6 ± 13.3	69.6 ± 11.9	81.8 ± 10.9 *	80.8 ± 14.9

Results are expressed as mean ±S.D. for each group rats (*n* = 8); * *p* < 0.05 versus HF; ^#^ *p* < 0.05 versus HF + CO.

**Table 6:** The change of intestinal disaccharidase activities and fecal mucinase and β-glucuronidase activities in rats fed the different experimental diets for 10 weeks.

Diet	NC	HF	HF + LC	HF + CO
Maltase (mg glucose/min/mg protein)	774.7 ± 124.4 *	986.8 ± 73.2	837.6 ± 128.6 *^,#^	990.2 ± 30.9
Lactase (mg glucose/min/mg protein)	152.2 ± 50.7 *	205.1 ± 31.6	163.5 ± 41.9 *	184.7 ± 18.4
Sucrase (mg glucose/min/mg protein)	217.6 ± 66.4 *	329.3 ± 62.2	228.2 ± 103.6 *	266.2 ± 59.4
Mucinase (nmole reducing sugar/min/mg protein)	3.6 ± 0.9 *	4.9 ± 1.0	4.0 ± 1.1	3.1 ± 0.6 *
β-glucuronidase (nmole phenolphthalein/min/mg protein)	2.4 ± 0.4 *	3.1 ± 0.5	2.2 ± 0.7 *	2.5 ± 0.2 *

Results are expressed as mean ±S.D. for each group rats (*n* = 8); * *p* < 0.05 versus HF; ^#^ *p* < 0.05 versus HF + CO.

## Abbreviations

CO: chitosan oligosaccharide; DM: diabetes mellitus; FAS: fatty acid synthetase; HF: high fat; HOMA-IR: homeostasis model assessment equation for insulin resistance; IRS: insulin receptor substrate; LC: low molecular weight chitosan; NC: standard rodent diet-fed rats; STZ: streptozotocin; T2DM: type 2 diabetes mellitus; TC: total cholesterol; TG: triglyceride; TNF-α: tumor necrosis factor-α.
